# Orphan nuclear receptor SHP regulates iron metabolism through inhibition of BMP6-mediated hepcidin expression

**DOI:** 10.1038/srep34630

**Published:** 2016-09-30

**Authors:** Don-Kyu Kim, Yong-Hoon Kim, Yoon Seok Jung, Ki-Sun Kim, Jae-Ho Jeong, Yong-Soo Lee, Jae-Min Yuk, Byung-Chul Oh, Hyon E. Choy, Steven Dooley, Martina U. Muckenthaler, Chul-Ho Lee, Hueng-Sik Choi

**Affiliations:** 1 National Creative Research Initiatives Center for Nuclear Receptor Signals and Hormone Research Center, School of Biological Sciences and Technology, Chonnam National University, Gwangju, Republic of Korea; 2 Korea Research Institute of Bioscience and Biotechnology, University of Science and Technology (UST), Yuseong-gu, Daejeon, Republic of Korea; 3 Department of Microbiology, Chonnam National University Medical School, Gwangju, Republic of Korea; 4 Department of Medical Science and Infection Biology, Chungnam National University School of Medicine, Daegeon, Republic of Korea; 5 Lee Gil Ya Cancer and Diabetes Institute, Gachon University Graduate School of Medicine, Incheon, Republic of Korea; 6 Department of Medicine II, Section Molecular Hepatology, Medical Faculty Mannheim, Heidelberg University, Mannheim, Germany; 7 Department of Pediatric Oncology, Hematology and Immunology, University of Heidelberg, Heidelberg, Germany; 8 Molecular Medicine Partnership Unit, University of Heidelberg, Heidelberg, Germany

## Abstract

Small heterodimer partner (SHP) is a transcriptional corepressor regulating diverse metabolic processes. Here, we show that SHP acts as an intrinsic negative regulator of iron homeostasis. SHP-deficient mice maintained on a high-iron diet showed increased serum hepcidin levels, decreased expression of the iron exporter ferroportin as well as iron accumulation compared to WT mice. Conversely, overexpression of either SHP or AMP-activated protein kinase (AMPK), a metabolic sensor inducing SHP expression, suppressed BMP6-induced hepcidin expression. In addition, an inhibitory effect of AMPK activators metformin and AICAR on BMP6-mediated hepcidin gene expression was significantly attenuated by ablation of SHP expression. Interestingly, SHP physically interacted with SMAD1 and suppressed BMP6-mediated recruitment of the SMAD complex to the hepcidin gene promoter by inhibiting the formation of SMAD1 and SMAD4 complex. Finally, overexpression of SHP and metformin treatment of BMP6 stimulated mice substantially restored hepcidin expression and serum iron to baseline levels. These results reveal a previously unrecognized role for SHP in the transcriptional control of iron homeostasis.

The hepatic peptide hormone hepcidin coordinates body iron homeostasis that is maintained by the tight regulation of dietary iron absorption and release of iron from macrophages and hepatocytes^1^. Hepcidin expression in the liver is up-regulated by iron excess and inflammatory cues, causing a reduction of serum iron levels. In contrast, iron deficiency, hypoxia, and increased erythropoetic activity down-regulates hepcidin, leading to increased systemic iron availability. Hepcidin binds to ferroportin (FPN), an iron exporter present on the membrane of enterocytes, macrophages, and hepatocytes, and promotes its internalization and lysosomal degradation^2^. Decreased FPN in response to hepcidin thus inhibits iron recycling from senescent erythrocytes by macrophages, dietary iron uptake in duodenal enterocytes, and iron mobilization from hepatic stores.

Two major signaling pathways regulate hepcidin expression. The hepcidin response to inflammation mediated by stimuli, such as interleukin-6 (IL-6), requires activation of Janus kinase (JAK)/signal transducer and activator of transcription 3 (STAT3) signaling in hepatocytes^3^,^4^. The hepcidin promoter contains a conserved STAT3-binding site, which is critical for responding to inflammation mediated stimulation. In addition, lipopolysaccharide (LPS) treatment of mice triggers induction of hepcidin via JAK/STAT3 activation. The resulting hypoferremia is part of the innate immune response that protects the host from infections^1^. The iron-response of hepcidin is further regulated by bone morphogenetic protein (BMP)-SMAD signaling^5^,^6^,^7^. BMP6 binds to type I and type II BMP receptors (BMPR-I and BMPR-II) in the presence of the BMP coreceptor hemojuvelin (encoded by *HFE2*) and induces phosphorylation of Receptor (R)-SMAD proteins (SMAD1, SMAD5 and SMAD8)^8^. Subsequently, activated R-SMADs form heteromeric complexes with SMAD4, called the co-SMAD, which translocate to the nucleus to regulate hepcidin expression at the transcription level.

Small heterodimer partner (SHP; NR0B2) is an atypical orphan member of the nuclear receptor superfamily that lacks the conventional DNA binding domain^9^. However, it is classified as a nuclear receptor due to the presence of a putative ligand-binding domain. SHP is predominantly expressed in the liver, and acts as a transcriptional corepressor of a variety of nuclear receptors and transcription factors implicated in various metabolic processes, such as glucose, lipid and bile acid metabolism^10^,^11^,^12^. In addition, SHP is upregulated by AMP-activated protein kinase (AMPK), an energy sensor and a metabolic switch factor. Previously, we demonstrated that SHP gene expression is strongly induced by AMPK activators such as metformin, sodium arsenite, hepatocyte growth factor, and macrophage-stimulating factor, leading to inhibition of hepatic gluconeogenesis^13^,^14^,^15^. In spite of considerable progress in understanding the functions of SHP in diverse physiological processes, a role of SHP in iron metabolism has not yet been elucidated. In the current study, we describe SHP as transcriptional corepressor of BMP6-mediated hepcidin expression that is triggered by an iron signal.

## Results

### SHP deficiency affects hepcidin gene expression in liver of mice exposed to high-iron diet

**Figure 1:**
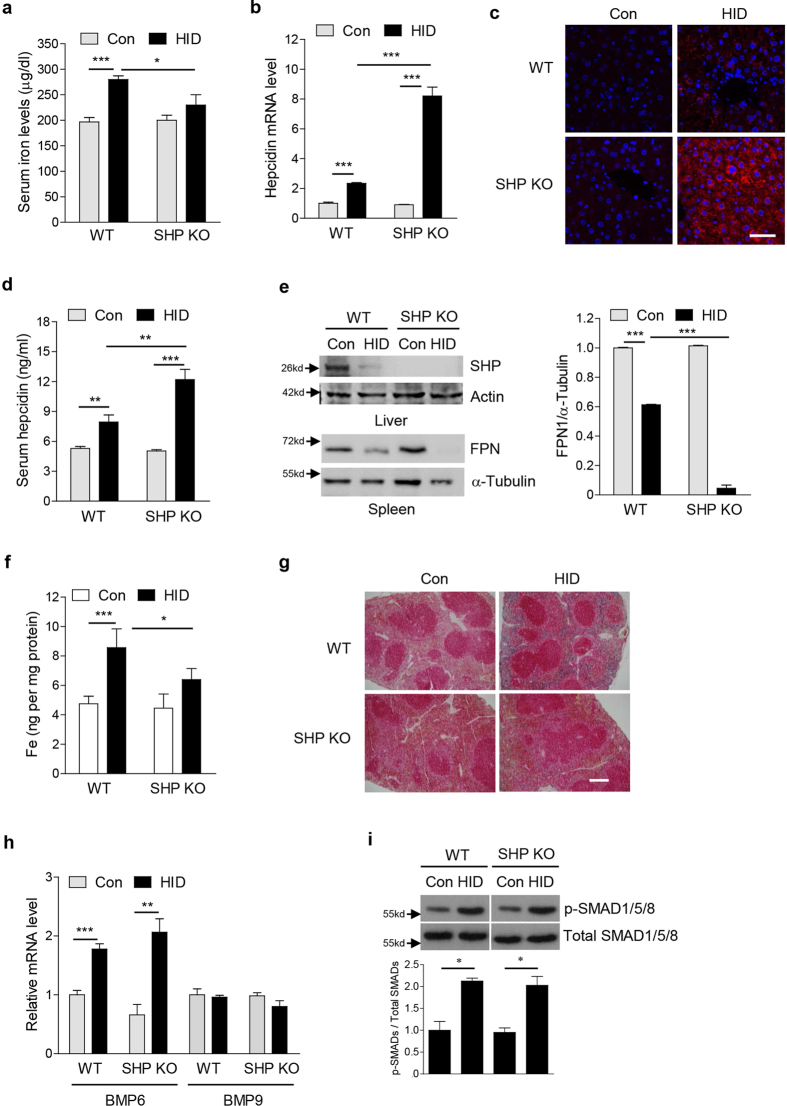
SHP deficiency alters hepcidin gene expression in liver of HID mice. (**a**–**i**) WT and SHP KO mice (*n* = 5 per group) were fed with high-iron diet (HID, 8 g/kg) for 3 weeks. (**a**) Serum iron level. (**b**) Hepcidin mRNA level in liver. (**c**) Hepcidin expression in mouse liver. IHC was performed using an antibody against hepcidin. Scale bar shows 50 μm. (**d**) Serum hepcidin level. (**e**) Western blot analysis (left panel) showing hepatic SHP and splenic FPN expression and graphical representation (right panel) showing splenic FPN expression. (**f**) Splenic iron level. (**g**) Perls’ prussian blue staining in spleen. Scale bar shows 200 μm. (**h**) BMP6 and BMP9 mRNA levels in liver. (**i**) Western blot analysis (top) and graphical representation (bottom) showing SMAD1/5/8 phosphorylation in liver. The grouping of the images is from different parts of the same gel. Data are presented as means ± SD. Arrows show locations of molecular weight markers. The experiment was repeated on a minimum of three separate occasions. The western blot images were cropped with a grey cropping line. All gels for western blot analysis were run under the same experimental conditions. *P < 0.05, **P < 0.01, ***P < 0.001 by two-tailed Student *t*-test.

To examine whether SHP alters systemic iron homeostasis, SHP KO mice and C57BL/6J mice were maintained on a high-iron diet (HID) for 3 weeks and subsequently analyzed for iron related parameters. As expected, serum iron levels were higher in HID-fed WT mice compared to control mice maintained on a regular diet (Fig. 1a). Interestingly, SHP KO mice maintained on a HID showed significantly lower serum iron levels than corresponding WT mice. This may be explained by significantly higher hepatic hepcidin mRNA expression and serum hepcidin levels in SHP KO mice compared to WT mice (Fig. 1b–d). Hepcidin binds to the iron exporter FPN to control its degradation. Consistently, SHP KO mice maintained on a HID showed decreased splenic FPN protein levels compared to WT mice (Fig. 1e). Moreover, splenic iron levels were much less in SHP KO mice maintained on a HID (Fig. 1f,g). However, SHP deficiency did not affect iron-mediated BMP6 gene expression and SMAD1/5/8 activation in the liver compared to control (Fig. 1h,i). Although BMP9, a BMP family member primarily expressed and secreted by the liver, can also induce hepcidin expression^16^, BMP9 gene expression is not significantly changed in the liver of HID-fed WT mice compared to control mice maintained on a regular diet (Fig. 1h). These results suggest that BMP6 and SHP are involved in iron-mediated regulation of hepcidin expression in mouse liver.

### SHP inhibits BMP6-mediated hepcidin expression

**Figure 2:**
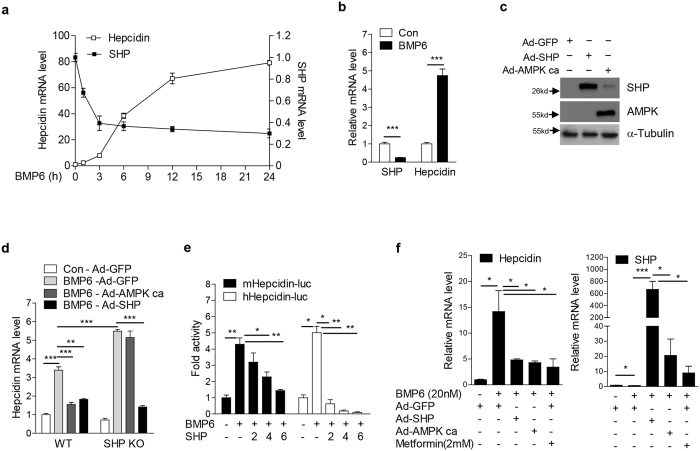
SHP suppresses BMP6-induced hepcidin gene expression. (**a**) Q-PCR analysis showing hepcidin and SHP mRNA levels in AML12 cells treated with BMP6 (20 nM). (**b**) Q-PCR analysis showing hepcidin and SHP mRNA levels in mouse hepatocytes treated with BMP6 (20 nM) for 24 h. (**c**) Western blot analysis, showing SHP and AMPK expression in HepG2 cells infected with Ad-GFP, Ad-Flag-SHP and Ad-AMPK ca for 24 h +, 10 MOI. (**d**) Q-PCR analysis showing hepcidin mRNA levels in hepatocytes isolated from wild-type and SHP knockout mice. Hepatocytes were treated with BMP6 (20 nM) and infected with Ad-GFP, Ad-AMPK ca or Ad-Flag-SHP for 24 h. (**e**) Inhibitory effect of SHP on BMP6-mediated induction of mouse and human hepcidin promoter activity. HepG2 cells were transfected with vectors expressing mouse Hepcidin-luc and mSHP or human Hepcidin-luc and hSHP, and treated with BMP6 (20 nM) for 24 h. 2, 200 ng; 4, 400 ng; 6, 600 ng. (**f**) Q-PCR analysis showing hepcidin (left panel) and SHP (right panel) mRNA levels in mouse primary hepatocytes treated with BMP6 or metformin and infected with Ad-GFP, Ad-AMPK ca or Ad-Flag-SHP for 24 h +, 10 MOI. Data are presented as means ± SD. Arrows show locations of molecular weight markers. The experiment was repeated on a minimum of three separate occasions. Western blot images were cropped with a grey cropping line. All gels for Western blot analysis were run under the same experimental conditions. *P < 0.05, **P < 0.01, ***P < 0.001 by two-tailed Student *t*-test.

BMP6 expression is increased in response to hepatic iron overload and a major contributor of iron-mediated hepcidin expression^6^. To identify possible links between SHP and hepcidin gene expression, AML12 cells were treated with BMP6. As expected, hepcidin mRNA expression was significantly increased by BMP6 stimulation for 1 h and was further enhanced at 24 h. In contrast, SHP mRNA expression was rapidly decreased 3 h after BMP6 treatment and maintained at reduced levels (Fig. 2a). Like in AML12 cells, SHP mRNA expression was also decreased in BMP6-treated primary murine hepatocytes, while hepcidin mRNA expression was increased (Fig. 2b), suggesting an inverse correlation between hepcidin and SHP gene expression upon BMP6 stimulation.

To demonstrate participation of SHP in BMP6-mediated hepcidin regulation, mouse hepatocytes isolated from WT and SHP KO mice were treated with BMP6 and infected with adenoviruses expressing SHP. To further examine whether or not AMPK, a strong inducer of SHP expression, is functionally linked to the regulation of BMP6-mediated hepcidin expression and may phenocopy the inhibitory effect of SHP, we transduced a constitutively active form of AMPK (Ad-AMPK ca) into hepatocytes. Overexpression efficiency of Ad-AMPK ca and Ad-SHP was confirmed in HepG2 cells (Fig. 2c). BMP6-mediated hepcidin gene expression was increased in primary hepatocytes isolated from SHP KO mice compared to WT mice (Fig. 2d). Furthermore, BMP6-mediated hepcidin response in WT hepatocytes was significantly decreased by overexpression of AMPK ca or SHP. In addition, the inhibitory effect of AMPK was almost entirely blunted in SHP-deficient hepatocytes, while the inhibitory effect of ectopically overexpressed SHP was maintained in SHP-deficient primary hepatocytes, comparable to WT hepatocytes. We next examined whether the repressor function of SHP on stimulation of hepcidin by BMP6 is conserved in HepG2 cells. We showed that SHP overexpression significantly inhibited both BMP6-induced mouse and human hepcidin promoter activity (Fig. 2e). The robustness of our findings were additionally confirmed in mouse primary hepatocytes, in that overexpression of SHP or AMPK ca and treatment with metformin decreased BMP6-mediated induction of hepcidin mRNA levels (Fig. 2f). Taken together, our data suggest that SHP indeed functions as a repressor of hepcidin transcriptional control by BMP6 signaling.

### Metformin inhibits BMP6-mediated hepcidin expression through SHP

**Figure 3:**
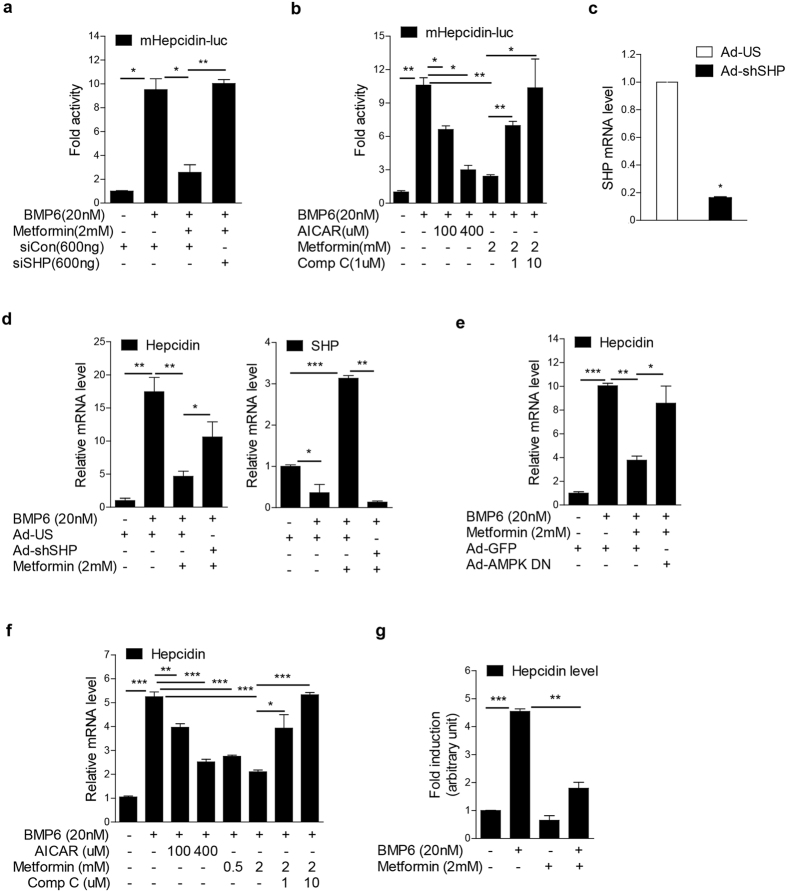
Metformin inhibits BMP6-mediated hepcidin gene expression through induction of SHP. (**a**) HepG2 cells were transfected with vectors expressing luciferase under the control of the hepcidin promoter, siControl or siSHP, and treated with BMP6 for 24 h, as indicated. (**b**) HepG2 cells were treated with BMP6, metformin, AICAR or Comp C for 24 h, as indicated in figure. (**c**) Q-PCR analysis showing SHP mRNA levels in AML12 cells infected with Ad-US and Ad-shSHP. (**d**) Q-PCR analysis showing hepcidin (left panel) and SHP (right panel) mRNA levels in AML12 cells. Cells were infected with Ad-US or Ad-shSHP and treated with BMP6 and metformin for 24 h +, 10 MOI. (**e**) Q-PCR analysis showing hepcidin mRNA levels in mouse primary hepatocytes. Cells were infected with Ad-GFP or Ad-AMPK DN and treated with BMP6 and metformin for 24 h +, 10 MOI. (**f**) Q-PCR analysis showing hepcidin mRNA levels in rat primary hepatocytes treated with BMP6 along with metformin, AICAR or compound C for 24 h. (**g**) Hepcidin levels in medium of cultured AML12 cells treated with BMP6 and metformin for 24 h. Data are presented as means ± SD. The experiment was repeated on a minimum of three separate occasions. *P < 0.05, **P < 0.01, ***P < 0.001 by two-tailed Student *t*-test.

Previously, we reported that metformin increases SHP gene expression through AMPK activation^13^,^14^,^15^. Therefore, based on the inhibitory effect of AMPK on BMP6-mediated hepcidin expression, we tested if metformin, an AMPK activator, affects BMP6 signaling-induced hepcidin expression. Indeed, metformin treatment suppressed the BMP6 effect on hepcidin promoter activity in HepG2 cells. This suppressive effect was attenuated by SHP knockdown and treatment with Comp C, an inhibitor of AMPK (Fig. 3a,b). We further demonstrated that activation of the hepcidin promoter by BMP6 was inhibited by treatment with AICAR, another AMPK activator (Fig. 3b).

Importantly, in AML12 cells and mouse primary hepatocytes, we confirmed the inhibitory effect on BMP6-mediated hepcidin gene expression by metformin, and showed that the inhibitory effect of metformin was significantly blunted by adenoviral overexpression of a short hairpin knock down construct targeting SHP (Ad-shSHP) (Fig. 3c,d) or of a dominant negative form of AMPK (Ad-AMPK DN) (Fig. 3e). In addition, we also confirmed the inhibitory effect of AICAR and metformin on BMP6-mediated hepcidin expression in rat hepatocytes, which was attenuated by treatment with Comp C (Fig. 3f). Finally, metformin significantly decreased hepcidin secretion upon BMP6 stimulation in AML12 cells (Fig. 3g). These results strongly suggested that the AMPK signal is connected to the regulation of iron metabolism through induction of SHP.

### SHP represses SMAD signaling activity via inhibition of DNA-binding of SMAD1 and SMAD4 complex

**Figure 4:**
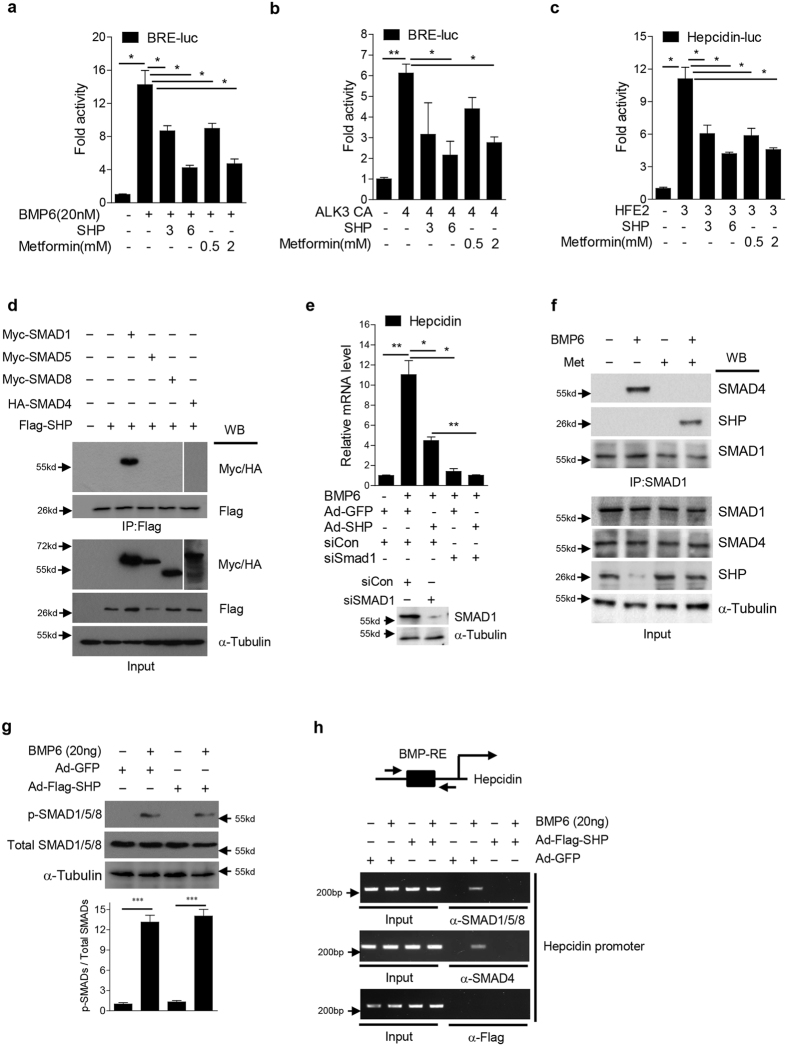
SHP represses BMP-mediated SMAD1 transactivation via inhibition of its DNA binding. (**a**,**b**) HepG2 cells were transfected with BRE-luc reporter plasmid along with vectors expressing SHP or ALK3 CA, and treated with BMP6 or metformin for 24 h. 3, 300 ng; 4, 400 ng; 6, 600 ng. (**c**) HepG2 cells were transfected with vectors expressing luciferase under the control of the hepcidin promoter, Hfe2 or SHP, and treated with metformin for 24 h. 3, 300 ng; 6, 600 ng. (**d**) Western blot analysis showing interaction of SHP and SMADs in 293T cells transfected with vectors expressing myc-SMAD1, myc-SMAD5, myc-SMAD8, HA-SMAD4 and Flag-SHP. The grouping of the image is from different parts of the same gel. (**e**) Q-PCR analysis showing hepcidin mRNA levels (top) and Western blot analysis showing SMAD1 expression (bottom) in mouse primary hepatocytes. Cells were transfected with siCon or siSMAD1 and infected with Ad-GFP or Ad-Flag-SHP. BMP6 was provided for 24 h+, 10 MOI. (**f**) Western blot analysis showing endogenous interaction of SHP and SMAD1 in HepG2 cells treated with BMP6 (20 nM) or metformin (2 mM) for 24 h. (**g**) Western blot analysis (top) and graphical representation (bottom) showing SHP effect on BMP6-medated SMAD1/5/8 phosphorylation. AML12 cells were infected with Ad-GFP or Ad-Flag-SHP (+10 MOI) and treated with BMP6 for 24 h. (**h**) ChIP assay was performed using soluble chromatin immunoprecipitated with anti-SMAD1/5/8, anti-SMAD4, and anti-Flag antibody. Mouse primary hepatocytes were treated with BMP6 for 24 h, and infected with Ad-GFP and Ad-Flag-SHP for 48 h. BMP-RE indicates BMP response element. Data are presented as means ± SD. Arrows show locations of molecular weight markers. The experiment was repeated on a minimum of three separate occasions. The western blot images were cropped with a grey cropping line. All gels for western blot analysis were run under the same experimental conditions. *P < 0.05, **P < 0.01 by two-tailed Student *t*-test.

It has been reported that dietary iron increases hepcidin expression through BMP6-mediated activation of SMAD1/5/8 signaling^6^. We transfected BRE-luciferase reporter (BRE-luc) constructs in HepG2 cells to investigate whether SHP would inhibit the BMP6-SMAD signaling pathway. Induction of BRE-luc activity by BMP6 stimulation or co-transfection of a constitutive active form of ALK3 (ALK3 CA), a type I BMP receptor, was significantly decreased by both co-transfection of SHP and treatment with metformin (Fig. 4a,b). Hemojuvelin (Hjv) (encoded by *HFE2*) acts as a BMP coreceptor to enhance hepcidin expression^17^. Indeed, Hjv expression led to a significant induction of hepcidin promoter activity as compared to the control, which was attenuated by both co-transfection of SHP and treatment with metformin (Fig. 4c). Taken together, these results indicate that SHP represses BMP signaling leading to SMAD1/5/8 transactivation.

To identify the molecular mechanism, by which SHP inhibits SMAD1/5/8 transactivation, we performed co-immunoprecipitation analysis in 293T cells transfected with vectors expressing SMADs and SHP. We found that SHP strongly interacts with SMAD1, but not with other SMADs (Fig. 4d). Based on these results, we knocked down SMAD1 in mouse primary hepatocytes infected with Ad-GFP or Ad-SHP, and treated with BMP. BMP6-mediated induction of hepcidin mRNA levels was blocked by SMAD1 knockdown, indicating that mainly SMAD1 is required for BMP6-mediated hepcidin expression (Fig. 4e). These results suggest that SHP exerts its effect on BMP6-mediated hepcidin expression predominantly through inhibition of SMAD1.

In addition, it is reported that BMP stimulation of hepcidin causes formation of a heteromeric complex between SMAD4 and phosphorylated SMAD1/5/8^8^,^18^. Therefore, we now examined whether SHP interferes with the formation of heteromeric complex between phosphorylated SMAD1 and SMAD4 in HepG2 cells treated with BMP6 or metformin. Indeed, SHP induction by metformin inhibited the BMP6-mediated the formation of heteromeric complex between SMAD1 and SMAD4 through its association with SMAD1 (Fig. 4f). However, adenoviral overexpression of SHP did not affect BMP6-mediated SMAD1/5/8 phosphorylation in AML12 cells (Fig. 4g). Therefore, to examine whether or not SHP decreases the DNA-binding ability of SMAD complexes to activate the hepcidin promoter, we performed ChIP assays in mouse hepatocytes that were treated with BMP6 and infected with Ad-Flag-SHP. BMP6 stimulation induced occupancy of the BRE on the hepcidin promoter by both R-SMADs and SMAD4, which was almost entirely abrogated by overexpression of SHP. However, SHP did not bind by itself to the BRE on the hepcidin promoter (Fig. 4h). These results suggest that SHP disrupts BMP6-mediated hepcidin expression by inhibiting SMAD1 and SMAD4 interaction and binding to the hepcidin promoter.

### SHP suppresses BMP6-mediated hepcidin expression in mice

**Figure 5:**
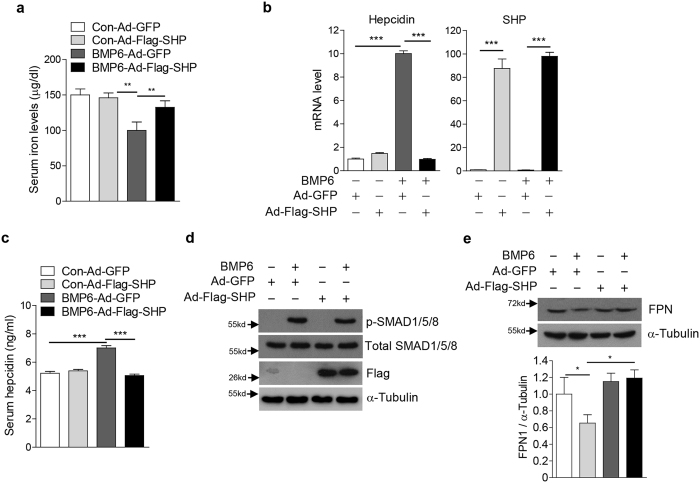
SHP abrogates the BMP6 effect on iron metabolism through inhibition of hepcidin gene expression in mice. (**a**–**d**) C57/BL6 mice were injected with Ad-GFP (*n* = 4 per group, 5.9 × 10^9^ pfu) or Ad-Flag-SHP (*n* = 5 per group, 5.9 × 10^9^ pfu) via the tail-vein, and treated with Vehicle or BMP6 (500 μg/kg, i.p.) for 6 h at day 5 after the infection. (**a**) Serum iron level. (**b**) Q-PCR analysis showing hepcidin and SHP mRNA levels in liver. (**c**) Serum hepcidin levels. (**d**) Western blot analysis showing SMAD1/5/8 phosphorylation and SHP expression in liver. (**e**) Western blot analysis showing FPN expression in spleen (top). Graphical representation showing FPN expression (bottom). Data are presented as means ± SD. Arrows show locations of molecular weight markers. The western blot images were cropped with a grey cropping line. All gels for western blot analysis were run under the same experimental conditions. **P < 0.01, ***P < 0.001 by two-tailed Student *t*-test.

**Figure 6:**
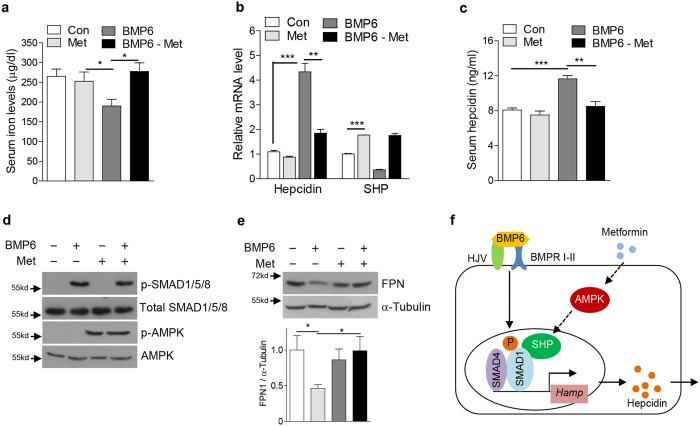
Metformin rescues BMP6-mediated alteration of iron metabolism in mice. (**a**–**e**) C57/BL6 mice (*n* = 4 per group) were treated with BMP6 (500 μg/kg, i.p.) and metformin (200 mg/kg, p.o.). (**a**) Serum iron level. (**b**) Q-PCR analysis showing hepcidin and SHP mRNA levels in liver. (**c**) Serum hepcidin levels. (**d**) Western blot analysis showing SMAD1/5/8 phosphorylation and FPN in liver. (**e**) Western blot analysis (top) and graphical representation (bottom) showing FPN expression in spleen. (**f**) Schematic diagram of SHP-mediated inhibition of BMP6-SMADs pathway. Data are presented as means ± SD. Arrows show locations of molecular weight markers. The western blot images were cropped with a grey cropping line. All gels for western blot analysis were run under the same experimental conditions. *P < 0.05, **P < 0.01, ***P < 0.001 by two-tailed Student *t*-test.

Based on our findings in cultured cells, we next aimed to determine whether SHP modulates BMP6-mediated alterations of iron metabolism through reduction of hepcidin expression *in vivo*. As expected, intraperitoneal injection of BMP6 into WT mice caused a significant induction of hepcidin levels in liver and serum, a reduction of FPN expression in the spleen as well as decreased serum iron levels (Fig. 5a–e). Adenoviral overexpression of SHP restored all alterations in iron homeostasis induced by BMP6 stimulation to basal levels. Consistent with the finding that metformin induces SHP expression^10^, we further showed that metformin treatment attenuates the BMP6-related changes of iron metabolism. Similar to ectopic SHP expression, metformin treatment blunted the BMP6-controlled hepcidin response, increased FPN expression in the spleen as well as serum iron levels (Fig. 6a–e). Taken together, these results suggest that SHP acts as a critical negative regulator for the BMP6-mediated induction of hepcidin, which causes physiological alterations of iron homeostasis.

## Discussion

Hepcidin plays an important role in coordinating systemic demand for iron with its acquisition. Hepcidin is expressed in the liver and its expression is mainly regulated by two major signaling pathways, the iron-controlled BMP6/SMAD and the inflammatory interleukin-6 (IL-6)-JAK-STAT3 pathways. Previously, it was demonstrated that orphan nuclear receptor SHP inhibits IL-6-mediated STAT3 activation through direct interaction of SHP and STAT3 in hepatocytes^13^. In addition, SHP was reported to act as a transcriptional corepressor of CCAAT/enhancer-binding protein alpha and estrogen-related receptor gamma, both involved in the transcriptional regulation of hepcidin expression^19^,^20^. In this study, we demonstrated that SHP is an intrinsic transcriptional repressor of SMAD1-activated hepcidin expression triggered by BMP6 signaling (Fig. 6f). We showed that (1) hepcidin and SHP mRNA expression inversely correlated upon BMP6 stimulation; (2) SHP-deficient mice produced more hepcidin when maintained on a HID; (3) SHP overexpression decreased BMP6-induced hepcidin expression in hepatocytes from WT and SHP KO mice, and that (4) the inhibitory effect of SHP is explained by inhibition of DNA binding of the SMAD1 and SMAD4 complex to the hepcidin promoter. These findings suggest that SHP expression is critical for maintenance of iron homeostasis.

A number of studies indicated that SHP acts as a transcriptional corepressor of other nuclear receptors and transcription factors involved in diverse metabolic pathways. In particular, SHP is a negative regulator of hepatic gluconeogenesis through inhibition of the CREB-CRTC2 pathway, a major signal transduction axis of this metabolic process^21^. We also demonstrated that SHP suppresses IL-6-mediated STAT3 activation, thereby causing hepatic insulin resistance^13^. Furthermore, SHP is a key molecule for the feedback regulation of bile acid metabolism by nuclear receptors^12^. AMPK controls energy balance by sensing the cellular energy status through its activation by alteration of the AMP:ATP ratio^22^. Pharmaceutical AMPK activators, such as metformin, an anti-diabetic drug, inhibit hepatic gluconeogenesis through induction of SHP. In the current study, we demonstrated that pharmaceutical activation of AMPK caused the reduction of BMP6-mediated hepcidin expression. This effect was attenuated by the ablation of endogenous SHP expression, suggesting that SHP is mediating the AMPK effect and thus plays a key role linking cellular energy signals with iron metabolism.

Recently, it was reported that the gluconeogenic signal during starvation increases hepcidin expression through PGC-1α/CREBH, whereby hepcidin was identified as a gluconeogenic sensor mediating alteration of iron metabolism^23^. Furthermore, cholestasis, caused by a disruption of bile flow, down regulated hepcidin expression through inhibition of the IL-6-STAT3 pathway^24^. In contrast, it was reported that iron affects production of adiponectin, an adipokine produced by adipocytes^25^,^26^. Adiponectin, as an AMPK activator, is causally linked to insulin sensitivity. Iron is also associated with lipid metabolism in adipocytes because of the requirement for iron in the oxidation of lipids^27^. More recently, it was reported that dietary iron affects circadian glucose metabolism through heme synthesis^28^. Therefore, the causal relationship of SHP in connecting metabolic signals and iron metabolism needs to be further characterized.

The BMP6-SMAD1/5/8 pathway is critical for regulating hepcidin synthesis in response to dietary iron^6^. Although a number of studies defined the role of BMP6 in the regulation of iron metabolism, details of the molecular mechanism by which dietary iron increases BMP6 expression is unclear. In response to serum iron, BMP expression is mainly upregulated in hepatic stellate cells (HSCs), suggesting that BMP6 acts in a paracrine manner to influence hepcidin transcription in neighboring hepatocytes^29^. In this study, we also found that WT mice fed with HID showed induction of BMP6 gene expression in the liver compared to control mice. SHP deficiency did not significantly affect high iron-mediated BMP6 expression and SMAD1/5/8 phosphorylation in the liver, suggesting that SHP is not affecting BMP6 expression in HSCs and the paracrine action of BMP6 to hepatocytes. Finally, we demonstrated that the inhibitory effect of SHP was mainly mediated through inhibition of SMAD1 and SMAD4 DNA-binding to the hepcidin promoter.

In summary, we identified a role of SHP as negative regulator for BMP6-mediated hepcidin expression in cultured cells and mice as well as molecular mechanisms active in this process. These findings indicate that the targeting of SHP by pharmacological agents may provide an attractive means for therapeutically controlling abnormal regulation of iron metabolism, leading to iron deficiency or iron overload.

## Materials and Methods

### Chemicals

BMP6 (R&D system), metformin (1,1-dimethylbiguanide hydrochloride; Sigma Aldrich Co.), 5-aminoimdazole-4-carboxamide-1-β-d-ribofuranoside (AICAR, Sigma Aldrich Co.), and Compound C (Comp C, Sigma Aldrich Co.) were dissolved in the manufacturer-recommended solvents.

### DNA and recombinant adenovirus construction

Mouse (−982/+84) and human (−2762 bp) hepcidin gene promoters were described previously^19^,^30^. pcDNA3-Flag-SHP, pcDNA3-Myc-human SMAD1, pcDNA3-Myc-mouse SMAD5, pcDNA3-Myc-rat SMAD8, pcDNA3-HA-human SMAD4, pSUPER-siControl, pSUPER-siSHP were described previously^31^,^32^. pCMV-SPORT6-Hfe2 was purchased from Korea Human Gene Bank, Medical Genomics Research Center, KRIBB, Korea. Ad-GFP, Ad-AMPK ca, Ad-AMPK DN, Ad-US (unspecific shRNA for control) and Ad-shSHP were described previously^10^. Ad-Flag-SHP was generated using the pAd-easy system as described previously^33^. All viruses were purified using CsCl_2_.

### Animal experiments

SHP knockout (KO) mice with a C57BL/6 background^34^ and wild-type (WT) C57BL/6 control (The Jackson Laboratory) mice were used for this study. Mice were housed in a temperature-controlled room (22 ± 2 °C) in a specific pathogen-free facility on a standard 12-hour light/dark cycle and were fed standard chow and water ad libitum. For a chronic iron diet study, male 4-week old WT and SHP KO mice (*n* = 5 per group) were fed with normal (200 mg/kg) and high-iron (8 g/kg) for 3 weeks. We performed adenoviral expression of GFP (*n* = 4 per group, 5.9 × 10^9^ pfu) and SHP (*n* = 5 per group, 5.9 × 10^9^ pfu) in C57BL/6J mice, and carried out intraperitoneal injection of BMP6 (500 μg/kg) for 6 h at day 5 after the infection. In addition, we also performed oral administration of metformin (200 mg/kg) into mice (*n* = 4 per group) for two days and intraperitoneal administration of BMP6 (500 μg/kg) for 6 h. All animal experiments were approved by the Institutional Animal Use and Care Committee of the Korea Research Institute of Bioscience and Biotechnology (KRIBB-AEC-14168). All experiments were performed in accordance with approved guidelines of KRIBB.

### Cull culture and transient transfection

Maintenance of HepG2 (human hepatoma cells), AML12 (mouse immortalized hepatocytes) and 293T (human embryonic kidney cells) cells was performed as mentioned previously^19^. In brief, HepG2 and 293T cells were maintained in Dulbecco’s modified Eagle’s medium (DMEM) supplemented with 10% fetal bovine serum (FBS) and antibiotics in a humidified atmosphere, containing 5% CO_2_ at 37 °C. AML12 cells were cultured with DMEM/F-12 medium supplemented with 10% FBS, insulin-transferrin-selenium, dexamethasone (40 ng/ml) and antibiotics in a humidified atmosphere, containing 5% CO_2_ at 37 °C. Transient transfections were performed with SuperFect (Qiagen) or Lipofectamine 2000 (Invitrogen), according to the manufacturer’s instructions. siControl and siSMAD1 were purchased from Qiagen (cat. SI00177072) and their transfections were carried out using Lipofectamine RNAiMAX (Invitrogen) according to the manufacturer’s instructions. BMP6, metformin, AICAR, Comp C treatment were performed as described in the figure legends.

### Culture of primary hepatocytes

Mouse and rat primary hepatocytes were isolated from C57BL/6J mice (male, 20–25 g) and Sprague-Dawley rats (male, 200–300 g) by collagenase perfusion as described previously^33^. Primary hepatocytes were maintained in M199 media (Cellgro) overnight for attachment. Adenoviral infections (Multiplicity of infection, MOI) and BMP6, metformin, AICAR, Comp C treatment were performed as described in figure legends.

### Quantitative PCR

Total RNAs were isolated from AML12 cells, primary hepatocytes or mice liver using the TRIzol reagent (Invitrogen), according to the manufacturer’s instructions. cDNAs were generated by Maxime RT PreMix Kit (iNtRON Biotechnology) and were analyzed by the Applied Biosystems StepOnePlus real-time PCR system (Applied Biosystems) using Power SYBR Green PCR Master Mix (Applied Biosystems). All data were normalized to L32 or actin expression.

### Chromatin Immunoprecipitation (ChIP) assay

The ChIP assay was performed according to the manufacturer’s protocol (Upstate). Briefly, mouse primary hepatocytes were treated with BMP6 and infected with Ad-GFP or Ad-Flag-SHP for 24 h. Cells were fixed with 1% formaldehyde and then harvested. Soluble chromatin was immunoprecipitated with anti-SMAD1/5/8 (Santa Cruz, cat. sc-6031-R), anti-SMAD1 (Cell Signaling, cat. 9743) and anti-Flag (for Flag-SHP) antibody. After recovering DNA, PCR was performed using primers encompassing the mouse Hepcidin promoter (forward 5′-GAGCCACAGTGTGACATCAC-3′, reverse 5′-GTCTAGGAGCCAGTCCCAGT-3′).

### Western blot analysis

50–100 μg protein from liver, spleen or cell lysates were separated by 10–12% SDS-PAGE, and then transferred to nitrocellulose membranes. Primary antibodies used for immunoblotting assays were against DYKDDDDK tag (FLAG, Cell Signaling, cat. 2368S), HA-tag (Cell Signaling, cat. 2367S), Myc-tag (Cell Signaling, cat. 2276), α-tubulin (Ab frontier, cat. LF-MA0117A), p-SMAD1/5/8 (Cell Signaling, cat. 9511S), SMAD1/5/8 (Santa Cruz, cat. sc-6031-R), p-AMPK (Cell Signaling, cat. 2535S), AMPK (Cell Signaling, cat. 2532S), SHP (Santa Cruz, cat. sc-30169), SMAD4 (Santa Cruz, cat. sc-7966), SMAD1 (Cell Signaling, cat. 9743) and Ferroportin 1 (Novus Biologicals, cat. NBP1-21502).

### Histomorphological analysis

Mouse tissues were processed in paraffin after fixation in 10% neutral buffered formalin. Sections of liver and spleen were stained with Perl’s Prussian blue to detect iron, and the nucleus was counterstained with neutral red. Sample analyses were performed using an optical microscope (BX51; Olympus, Tokyo, Japan). For immunofluorescence staining of hepcidin, liver sections were subjected to microwave antigen retrieval and then incubated overnight at 4 °C with anti-hepcidin antibody (1:100; abcam, Cambridge, MA, USA). Alexa Fluor 555-nm anti-rabbit antibody (1:200; Molecular probes, Eugene, OR, USA) was then applied for 1 hour at room temperature in the dark. Following each step, samples were analyzed by a confocal microscope (FV1200; Olympus, Tokyo, Japan).

### Serum iron and hepcidin measurement

All blood samples were taken by intracardiac puncture of mice under anesthesia before killing. Serum iron was measured using a spectrophotometric method (TBA-200FR NEO). Serum hepcidin was measured using a mouse hepcidin (Hepc) ELISA kit (CUSABIO, cat. CSB-E14395m) according to the manufacturer’s instructions.

### Iron measurement in tissue

Spleens were homogenized using a tissue Lyzer (BD Bioscience) after addition of 1 ml physiologic saline. The solution was centrifuged and the supernatant was used for quantification of iron. We determined total iron and protein concentration by a Beckman Coulter AU480 automatic biochemistry analysis system (Model AU-480). We calculated the average iron concentration in spleen by dividing total mean values over total protein contents.

### Statistical analysis

Data are expressed as means ± standard deviation (SD). Statistical analysis was performed using the two-tailed Student’s *t*-test. Differences were considered statistically significant at *p* < 0.05.

## Additional Information

**How to cite this article**: Kim, D.-K. *et al.* Orphan nuclear receptor SHP regulates iron metabolism through inhibition of BMP6-mediated hepcidin expression. *Sci. Rep.* **6**, 34630; doi: 10.1038/srep34630 (2016).

## References

[b1] Ganz T. & Nemeth E. Iron homeostasis in host defence and inflammation. Nat Rev Immunol 15, 500–510 (2015). 26160612 10.1038/nri3863 PMC4801113

[b2] Nemeth E. *et al.* Hepcidin regulates cellular iron efflux by binding to ferroportin and inducing its internalization. Science 306, 2090–2093 (2004). 15514116 10.1126/science.1104742

[b3] Wrighting D. M. & Andrews N. C. Interleukin-6 induces hepcidin expression through STAT3. Blood 108, 3204–3209 (2006). 16835372 10.1182/blood-2006-06-027631 PMC1895528

[b4] Verga Falzacappa M. V. *et al.* STAT3 mediates hepatic hepcidin expression and its inflammatory stimulation. Blood 109, 353–358 (2007). 16946298 10.1182/blood-2006-07-033969

[b5] Andriopoulos B. Jr. *et al.* BMP6 is a key endogenous regulator of hepcidin expression and iron metabolism. Nat Genet 41, 482–487 (2009). 19252486 10.1038/ng.335 PMC2810136

[b6] Kautz L. *et al.* Iron regulates phosphorylation of Smad1/5/8 and gene expression of Bmp6, Smad7, Id1, and Atoh8 in the mouse liver. Blood 112, 1503–1509 (2008). 18539898 10.1182/blood-2008-03-143354

[b7] Meynard D. *et al.* Lack of the bone morphogenetic protein BMP6 induces massive iron overload. Nat Genet 41, 478–481 (2009). 19252488 10.1038/ng.320

[b8] Casanovas G. *et al.* Bone morphogenetic protein (BMP)-responsive elements located in the proximal and distal hepcidin promoter are critical for its response to HJV/BMP/SMAD. J Mol Med (Berl) 87, 471–480 (2009). 19229506 10.1007/s00109-009-0447-2

[b9] Lee Y. S. *et al.* Structure and function of the atypical orphan nuclear receptor small heterodimer partner. Int Rev Cytol 261, 117–158 (2007). 17560281 10.1016/S0074-7696(07)61003-1

[b10] Kim Y. D. *et al.* Metformin inhibits hepatic gluconeogenesis through AMP-activated protein kinase-dependent regulation of the orphan nuclear receptor SHP. Diabetes 57, 306–314 (2008). 17909097 10.2337/db07-0381

[b11] Kim S. C. *et al.* All-trans-retinoic acid ameliorates hepatic steatosis in mice by a novel transcriptional cascade. Hepatology 59, 1750–1760 (2014). 24038081 10.1002/hep.26699 PMC4008145

[b12] Lu T. T. *et al.* Molecular basis for feedback regulation of bile acid synthesis by nuclear receptors. Mol Cell 6, 507–515 (2000). 11030331 10.1016/s1097-2765(00)00050-2

[b13] Kim Y. D. *et al.* Metformin ameliorates IL-6-induced hepatic insulin resistance via induction of orphan nuclear receptor small heterodimer partner (SHP) in mouse models. Diabetologia 55, 1482–1494 (2012). 22349108 10.1007/s00125-012-2494-4

[b14] Chanda D. *et al.* Sodium arsenite induces orphan nuclear receptor SHP gene expression via AMP-activated protein kinase to inhibit gluconeogenic enzyme gene expression. Am J Physiol Endocrinol Metab 295, E368–379 (2008). 18505831 10.1152/ajpendo.00800.2007

[b15] Chanda D. *et al.* Hepatocyte growth factor family negatively regulates hepatic gluconeogenesis via induction of orphan nuclear receptor small heterodimer partner in primary hepatocytes. J Biol Chem 284, 28510–28521 (2009). 19720831 10.1074/jbc.M109.022244 PMC2781394

[b16] Truksa J., Peng H., Lee P. & Beutler E. Bone morphogenetic proteins 2, 4, and 9 stimulate murine hepcidin 1 expression independently of Hfe, transferrin receptor 2 (Tfr2), and IL-6. Proc Natl Acad Sci USA 103, 10289–10293 (2006). 16801541 10.1073/pnas.0603124103 PMC1502450

[b17] Babitt J. L. *et al.* Bone morphogenetic protein signaling by hemojuvelin regulates hepcidin expression. Nat Genet 38, 531–539 (2006). 16604073 10.1038/ng1777

[b18] Wang R. H. *et al.* A role of SMAD4 in iron metabolism through the positive regulation of hepcidin expression. Cell Metab 2, 399–409 (2005). 16330325 10.1016/j.cmet.2005.10.010

[b19] Kim D. K. *et al.* Inverse agonist of estrogen-related receptor gamma controls Salmonella typhimurium infection by modulating host iron homeostasis. Nat Med 20, 419–424 (2014). 24658075 10.1038/nm.3483

[b20] Courselaud B. *et al.* C/EBPalpha regulates hepatic transcription of hepcidin, an antimicrobial peptide and regulator of iron metabolism. Cross-talk between C/EBP pathway and iron metabolism. J Biol Chem 277, 41163–41170 (2002). 12183449 10.1074/jbc.M202653200

[b21] Lee J. M. *et al.* AMPK-dependent repression of hepatic gluconeogenesis via disruption of CREB.CRTC2 complex by orphan nuclear receptor small heterodimer partner. J Biol Chem 285, 32182–32191 (2010). 20688914 10.1074/jbc.M110.134890 PMC2952219

[b22] Hardie D. G. AMPK–sensing energy while talking to other signaling pathways. Cell Metab 20, 939–952 (2014). 25448702 10.1016/j.cmet.2014.09.013 PMC5693325

[b23] Hentze M. W., Muckenthaler M. U., Galy B. & Camaschella C. Two to tango: regulation of Mammalian iron metabolism. Cell 142, 24–38 (2010). 20603012 10.1016/j.cell.2010.06.028

[b24] Huang Y. H. *et al.* Cholestasis downregulate hepcidin expression through inhibiting IL-6-induced phosphorylation of signal transducer and activator of transcription 3 signaling. Lab Invest 89, 1128–1139 (2009). 19652645 10.1038/labinvest.2009.82

[b25] Huang J. *et al.* Increased glucose disposal and AMP-dependent kinase signaling in a mouse model of hemochromatosis. J Biol Chem 282, 37501–37507 (2007). 17971451 10.1074/jbc.M703625200

[b26] Gabrielsen J. S. *et al.* Adipocyte iron regulates adiponectin and insulin sensitivity. J Clin Invest 122, 3529–3540 (2012). 22996660 10.1172/JCI44421 PMC3461897

[b27] Simcox J. A. & McClain D. A. Iron and diabetes risk. Cell Metab 17, 329–341 (2013). 23473030 10.1016/j.cmet.2013.02.007 PMC3648340

[b28] Simcox J. A. *et al.* Dietary iron controls circadian hepatic glucose metabolism through heme synthesis. Diabetes 64, 1108–1119 (2015). 25315005 10.2337/db14-0646 PMC4375081

[b29] Enns C. A. *et al.* Increased iron loading induces Bmp6 expression in the non-parenchymal cells of the liver independent of the BMP-signaling pathway. PLoS One 8, e60534 (2013). 23565256 10.1371/journal.pone.0060534 PMC3615098

[b30] Mleczko-Sanecka K. *et al.* SMAD7 controls iron metabolism as a potent inhibitor of hepcidin expression. Blood 115, 2657–2665 (2010). 20040761 10.1182/blood-2009-09-238105

[b31] Suh J. H. *et al.* Orphan nuclear receptor small heterodimer partner inhibits transforming growth factor-beta signaling by repressing SMAD3 transactivation. J Biol Chem 281, 39169–39178 (2006). 17074765 10.1074/jbc.M605947200

[b32] Chanda D., Xie Y. B. & Choi H. S. Transcriptional corepressor SHP recruits SIRT1 histone deacetylase to inhibit LRH-1 transactivation. Nucleic Acids Res 38, 4607–4619 (2010). 20375098 10.1093/nar/gkq227 PMC2919721

[b33] Kim D. K. *et al.* Orphan nuclear receptor estrogen-related receptor gamma (ERRgamma) is key regulator of hepatic gluconeogenesis. J Biol Chem 287, 21628–21639 (2012). 22549789 10.1074/jbc.M111.315168 PMC3381127

[b34] Park Y. J. *et al.* Loss of orphan receptor small heterodimer partner sensitizes mice to liver injury from obstructive cholestasis. Hepatology 47, 1578–1586 (2008). 18393320 10.1002/hep.22196

